# The Basement Membrane

**Published:** 1842-09-03

**Authors:** 


*The Basement Membrane.*—It had been ascertained by Henle, that the wall of an uriniferous tube is composed of two elements—a transparent membrane and an epithelial covering; to Mr. Bowman is due the merit of having shown that these elementary parts exist wherever true mucous membrane is found, and that skin is similarly compounded.

The primary and most essential of these elements is the transparent membrane. It affords perhaps the simplest and most beautiful example of a membranous expansion which can be met with in the body. It forms the basis on which the epithelial element is deposited; and to it, the mucous membrane owes whatever cohesive power, strength, or other physical property it possesses. Hence, Mr. Bowman has given it the appropriate name of *basement membrane*. It is homogeneous, structureless, and perfectly transparent; the highest powers of the microscope cannot detect in it any definite arrangement of particles. The transparent tube that envelopes the fleshy elements of the primitive fasciculus of voluntary muscle, presents precisely the same character, as does the wall of the nerve tubule, the posterior layer of the cornea, and the capsule of the crystalline lens. The basement membrane does not admit vessels into its texture. They ramify on its deep or parenchymal surface, and are supported by the submucous cellular tissue, or by a soft cystoblastema, which seems to be derived from recently effused liquor sanguinis, and from which, probably, the basement membrane and the epithelium have their nutrition and growth.—*Ibid, from London Medical Gazette.*

